# Supplementary material for: Identification of Freezing-Responsive microRNAs and Their Targets in Chinese Jujube by Small RNA and Degradome Sequencing
Source: Int J Mol Sci. 2026 Sep 4;27(17):7912. doi: 10.3390/ijms27177912 (PMC13566890; doi:10.3390/ijms27177912)
Supplement: Supplementary file 1 [file ijms-27-07912-s001.zip › Supplementary Material S2/novel_miR_192.pdf]

Provisional ID	:	novel_miR_192
Score total	:	0.5
Score for star read(s)	:	-1.3
Score for read counts	:	0
Score for mfe	:	0.2
Score for randfold	:	1.6
Score for cons. seed	:	
Total read count	:	72
Mature read count	:	72
Loop read count	:	0
Star read count	:	0

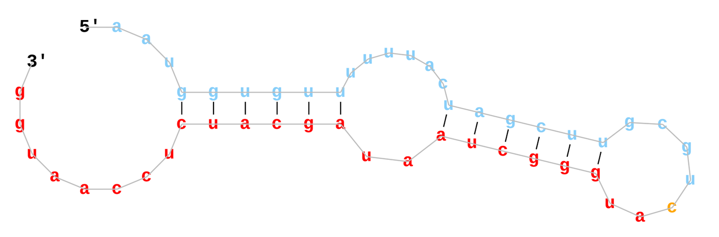

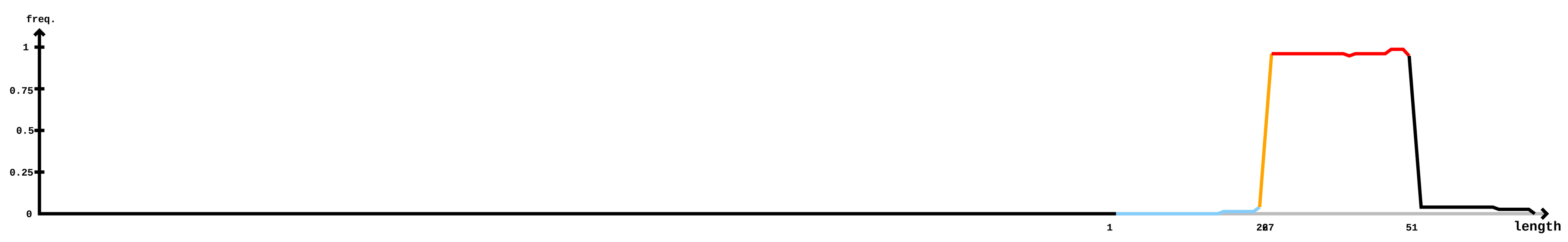

Star Mature

```
5'- gguauguucucccauacaauauuaauguccaucaaauuuaauuccagugaccacaccuuuuuauuucgguccaauuuauaauaccaauaauucuauugguaagagguuuuccauuuuuggguucuggauaugggcguuuaggauaaugugcagucaucuaauguaugauggcaaaaccaauggguguuuuuuacuagcuugcgucaugggcuaauagcaucuccaaugguuauguauauugauuuugacua -3'
    .(((((......)))))......((((((((.........(((((.((((...............)))).........((((((((....))))))))........((((....))))..))))).))))))))((((((((((((((((((((........))))))..(((((.(((.(....(((.((((((.......)))))).)))...).))).))))).)))))))).)))))))...
```

read	reads	mm	sample
cuugcgucaugggcuaauagc	1	0	JCK-1
ucaugggcuaauagcaucuccaauU	2	1	LT-2
augggcuaauagcaucuccaaug	1	0	LT-1
augggcuaauagcaucuccaaugg	5	0	LT-3
augggcuaauagcaucuccaaugg	15	0	JCK-2
augggcuaauagcaucuccaaugg	13	0	JCK-1
augggcuaauagcaucuccaaugg	5	0	LT-2
augggcuaauagcaucuccaaugg	10	0	JCK-3
augggcuaauagcaucuccaaugg	21	0	LT-1
ucuccaaugguuCuguauauugau	1	1	JCK-2
augguuauguauauugauuuugac	1	0	JCK-2
augguuauguauauugauuuugac	1	0	LT-3
